# Verifiable Differential Privacy Partial Disclosure for IoT with Stateless k-Use Tokens

**DOI:** 10.3390/s26041393

**Published:** 2026-02-23

**Authors:** Dachuan Zheng, Weijie Shi, Yilin Pan, Shengzhao Shu, Chunsheng Xu, Zihao Li, Bing Wang, Yuzhe Lin, Peishun Liu

**Affiliations:** Faculty of Information Science and Engineering, Ocean University of China, Qingdao 266100, China; 23020007169@stu.ouc.edu.cn (D.Z.); shiweijie@stu.ouc.edu.cn (W.S.); pyl@stu.ouc.edu.cn (Y.P.); shushengzhao@stu.ouc.edu.cn (S.S.); xcs@stu.ouc.edu.cn (C.X.); 2025lzh@stu.ouc.edu.cn (Z.L.); wb2230@stu.ouc.edu.cn (B.W.); 23020007071@stu.ouc.edu.cn (Y.L.)

**Keywords:** differential privacy, Internet of Things (IoT), partial disclosure, stateless tokens, verifiable computing

## Abstract

Internet of Things (IoT) applications often require only minimal necessary information—such as threshold judgments, binning, or prefixes—yet they must control privacy leakage arising from multi-round and cross-entity access without exposing raw values. Existing solutions, however, frequently rely on ciphertext structures and server-side states, making it difficult to define a leakage upper bound for restricted answers in the sense of Differential Privacy (DP), or they lack unified information budgeting and k-use control. To address these challenges, this paper proposes a verifiable differential privacy partial disclosure scheme for IoT. We employ DP accounting to uniformly constrain the leakage of three types of operators: threshold, binning, and prefix. Furthermore, we design stateless k-use tokens based on Verifiable Random Functions (VRFs) and chained receipts to generate publicly verifiable compliance evidence for each response. We implemented an end-edge-cloud prototype system and evaluated its performance on two use cases: smart meter threshold alarms and industrial sensor out-of-bound detection. Experimental results demonstrate that compared with a baseline relying on server-state counting for k-use control, our stateless k-use mechanism improves throughput by approximately 25–37% under concurrency scales of 1, 8, and 16, and reduces p95 latency by an average of 15%. Meanwhile, in multi-party splicing attack experiments, the re-identification accuracy remains stable in the 0.50–0.52 range, approximating random guessing. These results validate that the proposed scheme possesses low-energy engineering feasibility and audit-friendliness while effectively suppressing splicing risks.

## 1. Introduction

IoT systems are shifting from the centralized processing of uploaded raw data to cloud-edge collaborative computing [1], where terminals and edge nodes are required to provide only the minimal necessary information. Many applications do not require the raw values themselves but rather focus on whether a value crosses a specific threshold, falls into a discrete interval (binning), or matches a prefix rule. The rise of machine learning applications in IoT [2] has further increased the demand for privacy-preserving techniques. While differential privacy has been successfully applied to deep learning [3], adapting these techniques to resource-constrained IoT environments with verifiability requirements remains an open challenge.

To clarify the privacy protection scope discussed in this paper, we first briefly review related definitions. Differential Privacy (DP) [4] provides a rigorous mathematical frame-work that, by injecting an appropriate amount of noise into query results, probabilistically ensures that the presence or absence of a single record does not significantly alter the output distribution, thereby concealing individual characteristics. Local Differential Privacy (LDP) [5,6] further pushes this protection boundary to the data source, requiring that data be perturbed before leaving the device. This approach eliminates reliance on trusted third-party servers, ensuring that even in environments where servers are untrusted, attackers cannot infer users’ sensitive information from collected data.

However, without unified privacy accounting and verifiable evidence, such restricted responses—once spliced across different authorizations and multiple rounds—may still leak sensitive details. Furthermore, approaches relying on server-side persistent states or Trusted Execution Environments (TEEs) struggle to maintain long-term audit reproducibility in environments characterized by heterogeneous devices and unstable networks.

Several technical approaches focus on ciphertext-domain evaluation and access pattern control, yet critical limitations persist. In the domain of Order-Revealing/Preserving Encryption (ORE/OPE) and Property-Preserving Hashing (PPH), representative works [7,8,9,10] support comparison operations but suffer from cumulative order and boundary leakage under multi-round access. Similarly, Structured Searchable Encryption (SSE) schemes [11] consistently fail to conceal access patterns and result volumes. Research on leakage-abuse attacks [12,13] demonstrates that attackers can exploit these weaknesses via statistical approximation, highlighting the lack of “auditable and constrainable” leakage characterization in existing mechanisms.

Furthermore, a unified solution for Differential Privacy (DP) and access control remains elusive. Although Differential Privacy (DP) and Local Differential Privacy (LDP) mechanisms [5,6,14] are robust for statistical analysis, they struggle to represent discrete restricted operators (e.g., binning) and lack a unified budget model for multi-round auditability. Regarding access frequency limiting, existing schemes [15,16] typically rely on server-side persistent states or trusted hardware, making it difficult to maintain consistency across distributed edge and multi-cloud environments.

Finally, in the realm of verifiable computing [17], although systems like Pinocchio, PLONK (Permutations over Lagrange bases for Oecumenical Noninteractive arguments of Knowledge), and Verifiable Random Functions (VRFs) [18,19,20] provide mature tools for verifying single operators and randomness, they are insufficient for complex state-dependent scenarios. Existing works generally fail to provide a unified verification statement covering “mechanism consistency, budget deduction, and k-use limits,” effectively preventing the replayable auditing of multi-round restricted invocations.

Consequently, current research—both domestic and international—has not yet formed comprehensive solutions in the following three aspects: (1) controllable leakage boundaries under multiple authorizations and cross-entity splicing; (2) a unified information budget model for restricted operators such as ranges, binning, and prefixes; (3) k-use limits and verifiable evidence chains that function without server-side persistent states. These challenges constitute the primary motivation for the method proposed in this paper.

The main contributions of this paper are summarized as follows:

- We propose a verifiable differential privacy partial disclosure scheme for IoT data. Unlike a simple loose coupling of differential privacy accounting and API rate limiting, we atomically bind privacy budget consumption and k-use access control within a zero-knowledge proof relation. This design ensures that query proof generation must simultaneously satisfy dual constraints, thereby eliminating reliance on trusted server states, preventing separated attacks that bypass policies in a single dimension, and achieving full-process auditability.
- We present a unified system model and verification relation that encodes mechanism consistency and budget limits, enabling any party to independently audit unauthorized access or budget overdrafts via a publicly verifiable evidence path without accessing raw data.
- We implement a full-lifecycle prototype and demonstrate, through extensive performance evaluations and splicing attack simulations, that the system maintains acceptable constant-level engineering overheads and auditable risk bounds under practical parameters.

**Figure 1:**
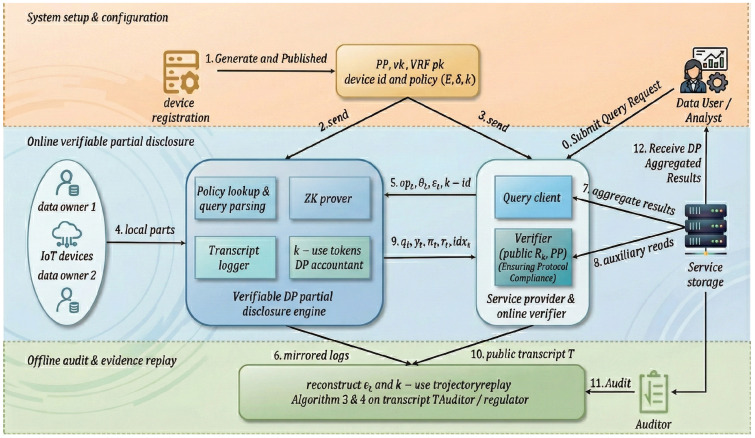
Overall architecture of the proposed verifiable DP partial-disclosure scheme for IoT data.

The remainder of this paper is organized as follows: Section 2 presents the system and threat models. Section 3 introduces preliminaries and notation conventions and formalizes the restricted response and privacy budget models. Section 4 describes the protocol design and core algorithms. Section 5 provides the correctness and security analysis. Section 6 presents the prototype implementation and experimental evaluation results. Finally, Section 7 concludes the paper and discusses future work. The overall architecture of the proposed scheme is illustrated in Figure 1.

## 2. System and Threat Model

We consider an IoT scenario where terminal devices act as the primary producers of raw data. To adapt to the dynamic nature of IoT data over time, this scheme employs a dynamic commitment mechanism. In each query round *t*, the device generates a new commitment $C_{i,t}=\mathrm{Com}\left(x_{i,t};\rho_{t}\right)$ for the current real-time observation value $x_{i,t}$. This mechanism ensures that the zero-knowledge proof constraints are always anchored to the current physical reading, thereby supporting effective verification of time-series data and avoiding the logical limitations of static commitments that cannot cover dynamic data. Edge nodes function solely for caching and request scheduling; they are assumed to be malicious and do not maintain any trusted counters or key states. Users initiate restricted queries to the devices, expecting to receive only restricted answers such as threshold judgments, discrete binning, or prefix disclosures. Independent verifiers or auditors can verify the consistency and compliance of each invocation based solely on commitments, zero-knowledge proofs (ZKPs), and chained receipts, without ever accessing the plaintext data or the server’s internal states. The overall system design eliminates reliance on server-side persistent states or TEEs.

Each data record binds a device identifier to a single scalar observation. Instead of raw values, the system returns restricted answers: determining whether a value crosses a given threshold, mapping the value to a preset discrete interval and returning the interval index, or outputting a fixed-length high-order prefix. During the registration phase, the device only generates identity-related keys and publishes the public keys as identity anchors; in each query round *t*, it independently samples the round’s randomness and generates corresponding evidence and meta-information to be provided along with $Trans_{t}$.

The security goals are fourfold: First, privacy accounting: Under multiple interactions regarding the same record, the system must satisfy the composition bound of (ε,δ)-DP, and the process must halt immediately upon budget exhaustion. Second, verifiability: A verifier, without accessing raw values, must be able to check that the current output is indeed computed from the specific commitment via the declared mechanism, that the privacy budget is not overdrawn, and that the invocation count has not exceeded its limit. Third, auditability: Receipts and proofs must be persistant and replayable, eliminating dependencies on historical session states. Fourth, source authenticity: The verifier must be able to confirm in a publicly verifiable manner that the data source $x_{i,t}$ participating in the zero-knowledge proof computation is indeed generated by device *i* holding a legitimate private key and has not been tampered with during transmission.

Regarding the threat model, we define edge nodes and cloud service providers as active attackers. This means that the above entities may not only behave as honest but also be curious to attempt inference but may also deviate from protocol specifications, actively attempt to tamper with receipt chains, forge zero-knowledge proofs, or replay historical credentials to undermine system integrity. Additionally, we assume that different authorized entities may collude, attempting to reconstruct original observations by sharing data. Therefore, the security of this scheme does not rely on the trustworthiness of the infrastructure but is enforced through cryptographic mechanisms (e.g., ZKP, VRF, and hash chains). To ensure source authenticity, this scheme introduces a digital signature mechanism. We assume that devices employ appropriate key protection mechanisms to ensure that stored private keys $\left(sk_{i},sk_{i}^{vrf}\right)$ are not leaked. Regarding physical side-channel attacks and source data authenticity issues, this protocol explicitly defines the security boundary: The protocol layer primarily guarantees the privacy and compliance (computational integrity) of the computational process, while key protection at the physical layer and anti-tampering of sensor data are assumed to be guaranteed by the underlying hardware root of trust. Specifically, we assume devices are equipped with industrial-standard hardware security modules (e.g., TPM 2.0 or Secure Element SE) for generating and storing identity private keys $\left(sk_{i},sk_{i}^{vrf}\right)$ and commitment randomness $\rho_{t}$ in an isolated environment. This hardware assumption ensures that even if an attacker physically accesses the device, it is difficult to extract long-term keys through differential power analysis (DPA), thereby providing a trusted anchor for the upper-layer zero-knowledge proof protocol.

The engineering constraints are defined as follows: Computation and proof generation on the device side must maintain a small, constant overhead. The edge node performs only queuing and throttling. Verification can be performed independently at any endpoint without reliance on a trusted time source. Randomness can be derived from a verifiable pseudo-random source. Invocation control employs stateless k-use tokens and generates verifiable receipts. During verification, these are checked alongside the privacy budget: The former constrains frequency and concurrency, while the latter constrains the upper bound of information disclosure.

## 3. Preliminaries, Notation and Formal Model

### 3.1. Notation and Public Parameters

The key mathematical notations, cryptographic parameters, and system variables used throughout this paper are summarized in Table 1.

### 3.2. Interfaces

#### 3.2.1. System Initialization

(1) $$\mathrm{Setup}\left(\lambda \right)\to PP$$

Given a security parameter λ, generate public parameters PP, which include the commitment system, hash function, VRF public key, and common reference string for zero-knowledge proofs. This step is typically performed offline by the system deployer.

#### 3.2.2. Device Registration

(2) $$\mathrm{RegisterDevice}\left(i,\varepsilon_{tot},k,PP\right)\to \left(pk_{i},pk_{i}^{vrf},\varepsilon_{0},rec_{0}\right)$$

When device *i* first joins the system, it runs a key generation algorithm to establish its digital identity. Specifically, the device generates a digital signature key pair $\left(pk_{i},sk_{i}\right)$ for source authentication and a Verifiable Random Function (VRF) key pair $\left(pk_{i}^{vrf},sk_{i}^{vrf}\right)$ for constructing unique session indices. Subsequently, the device publishes the public keys $pk_{i}$ and $pk_{i}^{vrf}$, along with the negotiated privacy budget parameters $\varepsilon_{tot}$ and invocation limit *k*, to the registration center as identity anchors. This process establishes the trust root for subsequent interactions without requiring any static data commitments during registration.

#### 3.2.3. Restricted Query

(3) $$\mathrm{Query}\left(i,t,x_{i,t},op_{t},\theta_{t},\varepsilon_{t-1},k_{i},rec_{t-1},sk_{i},sk_{i}^{vrf},pk_{i},pk_{i}^{vrf},PP\right)\to \left(Trans_{t},\varepsilon_{t},rec_{t}\right)$$

When the service party initiates the *t*-th query $q_{t}=\left(op_{t},\theta_{t}\right)$, the device first generates the restricted answer $y_{t}$ according to the specified differential privacy mechanism M and calculates the dynamic commitment $C_{i,t}=\mathrm{Com}\left(x_{i,t};\rho_{t}\right)$ for the current observation $x_{i,t}$. To ensure data source authenticity and non-repudiation, the device uses the private key $sk_{i}$ to digitally sign the tuple $\left(i,t,C_{i,t},y_{t},idx_{t},rec_{t}\right)$, generating $\sigma_{t}$. Finally, the device generates a zero-knowledge proof $\pi_{t}$ to prove that $y_{t}$ and the committed value $C_{i,t}$ satisfy the constraint relation of mechanism M and that privacy budget updates and k-use restrictions comply with regulations.

#### 3.2.4. Online Verification

(4) $$\mathrm{Verify}\left(Trans_{t},PP\right)\to \left\{0,1\right\}$$

The online verifier runs the verification algorithm (detailed in Algorithm 3) on input $Trans_{t}$ without accessing the raw $x_{i,t}$. If the output is 1, the invocation is considered compliant in all three dimensions (mechanism, budget, and k-use) and is recorded into the public transcript *T*; otherwise, the invocation is rejected.

### 3.3. Differential Privacy Mechanisms for Restricted Responses

This subsection presents local differential privacy mechanisms for three types of restricted responses op∈{thresh,bucket,prefix}, and specifies the single-round privacy cost $\Delta \varepsilon_{t}$ and budget update rules for each.

#### 3.3.1. Threshold Judgment

For threshold judgment operations, only answer “whether the current observation exceeds a given threshold.” Given threshold *T*, the true Boolean answer is

(5) $$b=1\left[x_{i},t\geq \tau \right]\in 0,1$$

The local mechanism uses a standard binary randomized response. Given privacy parameter $\varepsilon_{\mathrm{th}}>0$, the distribution of output $y_{t}\in \left\{0,1\right\}$ is

(6) $$\left\{\begin{array}{c} P\left[Y_{t}=b\right]=p,\\ \mathrm{Pr}\left[Y_{t}=1-b\right]=1-p,\\ p=e^{\left(\varepsilon_{t}h\right)}/\left(1+e^{\left(\varepsilon_{t}h\right)}\right) \end{array}\right.$$

This mechanism satisfies $\varepsilon_{\mathrm{th}}$-local differential privacy. The single-round privacy cost is

(7) $$\Delta \varepsilon_{t}=\varepsilon \left(o_{t}=thresh,\theta_{t}\right)=\varepsilon_{t}h$$

#### 3.3.2. Discrete Binning

For binning operations, we partition the observation domain *X* into a set of disjoint intervals:

(8) $$I_{1},\ldots ,I_{m}\subseteq X,\;\;\overset{m}{\underset{j=1}{⋃}}I_{j}=X,I_{j}\cap I_{j^{\prime }}=\emptyset \left(j\neq j^{\prime }\right)$$

Only answer “which interval the current observation falls into.” Let the true bucket index be

(9) $$j_{t}=bucket\left(x_{i},t;\theta_{t}\right)\in 1,\ldots ,m$$

where $\theta_{t}$ contains the specific bucket partition.

The local mechanism uses m-ary randomized response. Given privacy parameter $\varepsilon_{\mathrm{bk}}>0$, the distribution of output $y_{t}\in \left\{1,\ldots ,m\right\}$ is

(10) $$Pr\left[y_{t}=y\right]=\left\{\begin{array}{cc} \frac{e^{\varepsilon_{bk}}}{e^{\varepsilon_{bk}}+m-1}, & \;y=j_{t}\\ \frac{1}{e^{\varepsilon_{bk}}+m-1}, & \;y\neq j_{t} \end{array}\right.$$

This satisfies $\varepsilon_{\mathrm{bk}}$-LDP. The single-round privacy cost is

(11) $$\begin{array}{cc} \Delta \varepsilon_{t} & =\varepsilon \left({\mathrm{op}}_{t},\theta_{t}\right)=\varepsilon_{\mathrm{pre}},\\ {\mathrm{op}}_{t} & =\mathrm{bucket} \end{array}$$

#### 3.3.3. Prefix Disclosure

Prefix disclosure targets identifier-like fields (e.g., anonymized IDs, hash values), exposing only the high-order prefix without revealing the complete identifier. Let the original identifier be

(12) $$u=\left(c_{1},c_{2},\ldots ,c_{L}\right),\;c_{s}\in \Sigma$$

Only the prefix of length *ℓ*, $p=(c_{1},\ldots ,c_{\ell })$, is considered. The prefix space size is $m={\left|\Sigma \right|}^{\ell }$. Each prefix can be treated as a bucket index in {1,…,m}. The true prefix category is denoted $j_{t}\in \left\{1,\ldots ,m\right\}$. For consistency, the prefix mechanism also uses m-ary randomized response, but with an independent privacy parameter $\varepsilon_{\mathrm{pre}}>0$. The output distribution is

(13) $$\mathrm{Pr}\left[y_{t}=y\right]=\left\{\begin{array}{cc} \frac{e^{\varepsilon_{\mathrm{pre}}}}{e^{\varepsilon_{\mathrm{pre}}}+m-1}, & y=j_{t}\\ \frac{1}{e^{\varepsilon_{\mathrm{pre}}}+m-1}, & y\neq j_{t} \end{array}\right.$$

This distribution has the same form as Equation (8) and thus satisfies $\varepsilon_{\mathrm{pre}}$-LDP. The single-round privacy cost is

(14) $$\begin{array}{cc} \Delta \varepsilon_{t} & =\varepsilon \left({\mathrm{op}}_{t},\theta_{t}\right)=\varepsilon_{\mathrm{bk}},\\ {\mathrm{op}}_{t} & =\mathrm{prefix} \end{array}$$

**Engineering Trade-offs in Mechanism Selection:** For high-dimensional output space scenarios such as bucket and prefix queries, existing research shows that mechanisms like optimized local hash (OLH) typically provide better statistical utility than randomized response (RR) [21]. However, in a verifiable computing architecture, mechanism selection is constrained by the circuit complexity of zero-knowledge proofs. OLH relies on cryptographic hash functions, whose constraint count in arithmetic circuits is typically on the order of $10^{4}$, which would lead to a significant increase in proof generation time, making it difficult to adapt to resource-constrained IoT terminals. In contrast, the RR mechanism mainly involves lightweight Boolean comparisons and arithmetic operations, with extremely low circuit overhead. Experimental analysis was conducted in Section 6.8. Therefore, this scheme prioritizes RR to ensure system real-time performance and engineering feasibility.

#### 3.3.4. Budget Update and Accounting

For each device or record, let the initial budget be $\varepsilon_{\mathrm{tot}}$, and the balance before the *t*-th invocation be $\varepsilon_{t-1}$. After executing any of the above mechanisms, the budget is updated as

(15) $$\varepsilon_{t}=\varepsilon_{t-1}-\Delta \varepsilon_{t},\;\;0\leq \varepsilon_{t}\leq \varepsilon_{\mathrm{tot}}$$

If $\varepsilon_{t-1}<\Delta \varepsilon_{t}$, the current invocation should be rejected, and no $y_{t}$ or proof is generated.

Given a history of invocations,

(16) $${\left(o_{j},\varepsilon \left(o_{j},\theta_{j}\right)\right)}_{j\leq t}$$

the differential privacy accountant

(17) $$\left(\varepsilon^{*},\delta^{*}\right)\leftarrow Acc\left({\left\{\left(o_{j},\varepsilon \left(o_{j},\theta_{j}\right)\right)\right\}}_{j\leq t}\right)$$

outputs a composition bound $\left(\varepsilon_{\mathrm{acc}},\delta_{\mathrm{acc}}\right)$, where $\varepsilon_{\mathrm{acc}}$ is the global ε upper bound and $\delta_{\mathrm{acc}}$ is the failure probability upper bound. The accountant can be implemented using basic composition [22], advanced composition, or Rényi-DP [23]; the externally auditable semantics depend only on whether the composition bound is derived from the same Acc.

### 3.4. Stateless k-Use and Receipt Chain

#### 3.4.1. VRF-Generated Session Index

At the *t*-th invocation, the DP engine uses VRF [20] to compute on input (i,t):

(18) $$\left(\pi_{t}^{\mathrm{vrf}},r_{t}\right)\leftarrow VRF.Eval\left(sk_{vrf},i,t\right)$$

The verifier checks using the public key:

(19) $$\mathrm{VRF}.\mathrm{Verify}(pk_{\mathrm{vrf}},i,t,r_{t},\pi_{t}^{\mathrm{vrf}})=1$$

The session index is constructed from the VRF output $r_{t}$:

(20) $$idx_{t}=H(i\|t\|r_{t})$$

which uniquely identifies the *t*-th invocation of device *i* in the public transcript. Since VRF output is unique and pseudorandom for a given input, the device cannot forge multiple “legitimate-looking” additional invocation records.

#### 3.4.2. Hash-Chained Receipts

Let the public initial receipt be $rec_{0}$. The public metadata for the *t*-th round is $meta_{t}$. We chain all accepted invocations via a hash chain:

(21) $$rec_{t}=H(rec_{t-1}\|idx_{t}\left\|meta_{t}\right)$$

Thus, each new record is locked onto the previous receipt; if any intermediate record is deleted, inserted, or modified, the subsequent receipts will change. An auditor can simply recompute this formula from the beginning to detect any rollback or tampering by the server.

Additionally, to address potential view-forking attacks in distributed verification environments—where malicious devices present inconsistent receipt chain branches to different verifiers—this protocol recommends a periodic global anchoring mechanism. The system can set a period (e.g., every *N* rounds or a fixed time window) to force devices to publish their latest chain head receipt $rec_{t}$ to a public bulletin board or blockchain smart contract. When verifying, in addition to checking the connectivity of the hash chain, verifiers also need to verify that on-chain records are consistent with public anchors. This preserves the high performance of local verification while eliminating forking threats through eventual consistency.

#### 3.4.3. Stateless k-Use Counting

Since each accepted invocation produces a unique index $idx_{t}$ and is linked into the receipt chain, an auditor can simply extract all $idx_{t}$ entries for a device *i* from the public transcript *T* and count them to determine whether the upper limit *k* has been exceeded. The entire process does not rely on a server-maintained invocation counter, thus achieving stateless k-use limitation.

### 3.5. Unified Verification Relation

This subsection compresses three conditions—correct mechanism output, non-overdrawn budget, and adherence to k-use limits—into a unified verification relation *R*. Each round generates a zero-knowledge proof $\pi_{t}$ for independent checking by online or offline auditors.

#### 3.5.1. Public Input and Witness

For the *t*-th record, the public input vector is defined as

(22) $$inp_{t}=\left(i,C_{i,t},o_{t},\theta_{t},y_{t},\Delta \varepsilon_{t},\varepsilon_{t},k,idx_{t},rec_{t}\right)$$

The witness vector is

(23) $$wit_{t}=\left(x_{i,t},\rho_{t},\eta_{t},\varepsilon_{t-1}\right)$$

where $x_{i,t}$ is the raw observation of the current round, $\rho_{t}$ is the temporary randomness (fresh randomness) used to generate the dynamic commitment $C_{i,t}$, $\eta_{t}$ is the internal randomness of the DP mechanism, and $\varepsilon_{t-1}$ is the budget balance before updating. Note that the proof generation process uses $\rho_{t}$ rather than the static randomness from registration. This ensures that even if $x_{i,t}$ has the same value in different rounds, its commitment $C_{i,t}$ is statistically indistinguishable, preventing equality leakage in the ciphertext state.

#### 3.5.2. Three Sub-Predicates

The unified relation consists of the conjunction of three sub-predicates:

1. Mechanism consistency $R_{\mathrm{mech}}$: requires consistency between the dynamic commitment and mechanism invocation:

(24) $$C_{i,t}=Com\left(x_{i,t};\rho_{t}\right)$$

and $y_{t}$ is generated from $x_{i,t}$ via the specified DP mechanism.

2. Budget deduction consistency $R_{\mathrm{budget}}$: requires that the budget update follow the rules:

(25) $$\varepsilon_{t}=\varepsilon_{t-1}-\Delta \varepsilon_{t},\;\;\varepsilon_{t}\geq 0$$

and $\Delta \varepsilon_{t}=\varepsilon \left(o_{t},\theta_{t}\right)$ is consistent with the three types of mechanism parameters.

3. Invocation limit and receipt consistency $R_{\mathrm{kuse}}$: requires correct VRF and receipt chain and that k-use is not exceeded:

(26) $$\left\{\begin{array}{c} \mathrm{VRF}.\mathrm{Verify}(pk_{\mathrm{vrf}},i,t,\pi_{t}^{\left(\mathrm{vrf}\right)},r_{t})=1\\ idx_{t}=H\left(i∥t∥r_{t}\right)\\ {\mathrm{rec}}_{t}=H\left({\mathrm{rec}}_{t-1}∥idx_{t}∥{\mathrm{meta}}_{t}\right) \end{array}\right.$$

and based on all indexes related to device *i* in the public transcript, the number of accepted invocations does not exceed *k*.

#### 3.5.3. Unified Relation and Proof Semantics

Combining the three sub-predicates, the unified verification relation is written as

(27) $$R\left(inp_{t},wit_{t}\right)\Leftrightarrow R_{\mathrm{mech}}\wedge R_{\mathrm{budget}}\wedge R_{\mathrm{kuse}}$$

The DP engine locally invokes the proof algorithm to generate

(28) $$\pi_{t}\leftarrow \mathrm{ZK}.\mathrm{Prove}\left(PP,R,inp_{t},wit_{t}\right)$$

The online verifier or offline auditor executes

(29) $$ZK.Verify\left(PP,R,inp_{t},p_{t}\right)\in \left\{0,1\right\}$$

If and only if b=1, the invocation is considered compliant in all three dimensions (mechanism execution, budget deduction, and k-use limits) and is written into the public transcript *T*. In engineering implementations, *R* can also be split into three sub-relations and proven separately, then aggregated; in this paper, we uniformly denote it as one relation and one proof $\pi_{t}$ for simplicity.

## 4. Protocol Design and Algorithms

Based on the formal model, this section details the protocol implementation to ensure mechanism consistency, stateless budget constraints, and independent auditability. Unlike traditional approaches that separate data publication from auditing, we integrate these operations into atomic processes to cryptographically bind query results with budget deductions and invocation indices, effectively preventing ex-post tampering or selective disclosure.

The system workflow initiates with the device registration phase, which establishes the necessary initial states and cryptographic commitments for participating entities. The detailed workflow is presented in Algorithm 1.

The Restricted Query process serves as the core component, simultaneously handling LDP sampling, budget updates, and evidence construction within a single atomic invocation. By utilizing VRFs and ZKPs to certify compliance before data release, it ensures that every query output is verifiable and strictly adheres to system regulations, as detailed in Algorithm 2.
**Algorithm 1** Register Device**Input:** 
Security parameter λ, Device ID *i*, Total budget $\varepsilon_{tot}$, Limit *k*, Public Parameters PP**Output:** 
Public keys $pk_{i},pk_{i}^{vrf}$, Initial state $\varepsilon_{0},rec_{0}$1:$\left(pk_{i},sk_{i}\right)\leftarrow \mathrm{Sign}.\mathrm{KeyGen}\left(1^{\lambda }\right)$2:$\left(pk_{i}^{vrf},sk_{i}^{vrf}\right)\leftarrow \mathrm{VRF}.\mathrm{KeyGen}\left(1^{\lambda }\right)$            ▹ Generate independent VRF keys3:$\varepsilon_{0}\leftarrow \varepsilon_{tot}$4:$rec_{0}\leftarrow 0^{256}$                       ▹ Initialize receipt chain5:Store $\left(sk_{i},sk_{i}^{vrf}\right)$ using secure mechanism6:Publish $\left(i,pk_{i},pk_{i}^{vrf},\varepsilon_{tot},k,rec_{0}\right)$7:**return** $pk_{i},pk_{i}^{vrf},\varepsilon_{0},rec_{0}$

**Algorithm 2** Query
**Input:** 


$i,t,x_{i,t},opt,\theta_{t},\varepsilon_{t-1},k,rec_{t-1},sk_{i},sk_{i}^{vrf},pk_{i},pk_{i}^{vrf},PP$

**Output:** 
Transmission package $Trans_{t}$, Updated state $\varepsilon_{t},rec_{t}$  1:

$\Delta \varepsilon_{t}\leftarrow \varepsilon_{opt,\theta_{t}}$

  2:**if** $\varepsilon_{t-1}<\Delta \varepsilon_{t}$ **then**  3:    **return** ⊥  4:
**end if**
  5:

$\eta_{t}\leftarrow_{R}{\left\{0,1\right\}}^{*}$

  6:

$y_{t}\leftarrow \mathrm{Mechanism}\left(x_{i,t},opt,\theta_{t},\eta_{t}\right)$

  7:

$\varepsilon_{t}\leftarrow \varepsilon_{t-1}-\Delta \varepsilon_{t}$

  8:

$\rho_{t}\leftarrow_{R}{\left\{0,1\right\}}^{\lambda }$

  9:$C_{i,t}\leftarrow \mathrm{Com}\left(x_{i,t};\rho_{t}\right)$       ▹ Dynamic Commitment10:

$r_{t},\pi_{t}^{vrf}\leftarrow \mathrm{VRF}.\mathrm{Eval}\left(sk_{i}^{vrf},i,t\right)$

11:

$idx_{t}\leftarrow H(i\|t\|r_{t})$

12:

$meta_{t}\leftarrow \left(opt,\theta_{t},\Delta \varepsilon_{t},\varepsilon_{t}\right)$

13:

$rec_{t}\leftarrow H(rec_{t-1}\|idx_{t}\left\|meta_{t}\right)$

14:$msg_{t}\leftarrow i\|t\|C_{i,t}\|y_{t}\left\|idx_{t}\right\|rec_{t}$      ▹ Sign context including *t*15:

$\sigma_{t}\leftarrow \mathrm{Sign}\left(sk_{i},msg_{t}\right)$

16:

$inp_{t}\leftarrow \left(i,t,pk_{i},pk_{i}^{vrf},C_{i,t},opt,\theta_{t},y_{t},\Delta \varepsilon_{t},\varepsilon_{t},k,idx_{t},rec_{t}\right)$

17:

$wit_{t}\leftarrow \left(x_{i,t},\rho_{t},\eta_{t},\varepsilon_{t-1}\right)$

18:

$\pi_{t}\leftarrow \mathrm{ZK}.\mathrm{Prove}\left(PP,R,inp_{t},wit_{t}\right)$

19:

$Trans_{t}\leftarrow \left(\sigma_{t},\pi_{t}^{vrf},r_{t},\pi_{t},inp_{t}\right)$

20:**return** $Trans_{t},\varepsilon_{t},rec_{t}$


For real-time validation, the online verification process remains stateless and lightweight, enabling any verifier to accept or reject invocations by sequentially checking the VRF output, receipt hash chain, and ZKP validity against public parameters without maintaining historical context. The procedure is shown in Algorithm 3.

Finally, the offline auditing mechanism performs global consistency checks by reconstructing budget consumption trajectories and invocation statistics solely from public transcripts. This enables third parties to independently detect violations such as overdrafts or chain breaks, guaranteeing long-term traceability without accessing device internal states, as outlined in Algorithm 4.
**Algorithm 3** Verify**Input:** 
$Trans_{t}=\left(\sigma_{t},\pi_{t}^{vrf},r_{t},\pi_{t},inp_{t}\right)$, PP**Output:** 
b∈{0,1}  1:Parse $inp_{t}$ as $\left(i,t,pk_{i},pk_{i}^{vrf},C_{i,t},opt,\theta_{t},y_{t},\Delta \varepsilon_{t},\varepsilon_{t},k,idx_{t},rec_{t}\right)$  2:$msg_{t}\leftarrow i\|t\|C_{i,t}\|y_{t}\left\|idx_{t}\right\|rec_{t}$  3:**Step 1: Source authenticity**  4:**if** $\mathrm{Sign}.\mathrm{Verify}(pk_{i},\sigma_{t},msg_{t})=0$ **then**  5:    **return** 0              ▹ Invalid signature  6:**end if**  7:**Step 2: Index uniqueness**  8:**if** $\mathrm{VRF}.\mathrm{Verify}(pk_{i}^{vrf},\left(i,t\right),r_{t},\pi_{t}^{vrf})=0$ **or** $idx_{t}\neq H(i\|t\|r_{t})$ **then**  9:    **return** 0           ▹ Invalid VRF proof or index10:**end if**11:**Step 3: Computational integrity**12:**if** $\mathrm{ZK}.\mathrm{Verify}(PP,R,inp_{t},\pi_{t})=0$ **then**13:    **return** 0               ▹ Invalid ZK proof14:**end if**15:**return** 1

**Algorithm 4** Audit (Replay)
**Input:** 


$PP,pk_{\mathrm{vrf}},T,Acc,{\left(\varepsilon_{i}^{\mathrm{tot}},k_{i}\right)}_{i},B$

**Output:** 
result, *S*  1:State$\left[i\right]\leftarrow \left(\varepsilon_{i}^{\mathrm{tot}},rec_{\mathrm{init}},0\right)$ for each *i*; BatchQueue←⌀; S←⌀  2:**for** each $record_{t}\in T$ **do**  3:    $\left(i,t,op_{t},\theta_{t},y_{t},\Delta \varepsilon_{t},idx_{t},rec_{t},meta_{t},\pi_{t},r_{t},\pi_{t}^{\mathrm{vrf}}\right)\leftarrow record_{t}$  4:    $\left(\varepsilon_{\mathrm{bal}},rec_{\mathrm{prev}},cnt\right)\leftarrow \mathrm{State}\left[i\right]$  5:    **if** $\mathrm{VRF}.\mathrm{Verify}(pk_{\mathrm{vrf}},\left(i,t\right),r_{t},\pi_{t}^{\mathrm{vrf}})=0$ **or** $idx_{t}\neq H(i∥t∥r_{t})$ **then**  6:        **return** $\left(\mathrm{fail},record_{t}\right)$  7:    **end if**  8:    **if** $rec_{t}\neq H(rec_{\mathrm{prev}}∥idx_{t}∥meta_{t})$ **or** $\Delta \varepsilon_{t}\neq \varepsilon \left(op_{t},\theta_{t}\right)$ **then**  9:        **return** $\left(\mathrm{fail},record_{t}\right)$10:    **end if**11:    $\varepsilon_{\mathrm{new}}\leftarrow \varepsilon_{\mathrm{bal}}-\Delta \varepsilon_{t}$12:    **if** $\varepsilon_{\mathrm{new}}<0$ **then**13:        **return** $\left(\mathrm{fail},record_{t}\right)$14:    **end if**15:    cnt←cnt+116:    **if** $cnt>k_{i}$ **then**17:        S←S∪{i}18:    **end if**19:    $inp_{t}\leftarrow \left(i,C_{i,t},op_{t},\theta_{t},y_{t},\Delta \varepsilon_{t},\varepsilon_{t},k_{i},idx_{t},rec_{t}\right)$20:    $\mathrm{Add}(inp_{t},\pi_{t})$ to BatchQueue; $\mathrm{State}\left[i\right]\leftarrow \left(\varepsilon_{\mathrm{new}},rec_{t},cnt\right)$21:    **if** |BatchQueue|≥B **then**22:        valid←ZK.BatchVerify(PP,R,BatchQueue)23:        **if** valid=0 **then**24:           **return** (fail,BatchQueue)25:        **end if**26:        BatchQueue←⌀27:    **end if**28:
**end for**
29:**if** Size(BatchQueue)>0 **and** ZK.BatchVerify(PP,R,BatchQueue)=0 **then**30:    **return** (fail,BatchQueue)31:
**end if**
32:**for** each device *i* **do**33:    $\left(\varepsilon^{*},\Delta^{*}\right)\leftarrow \mathrm{Acc}\left(\mathrm{History}\;\mathrm{of}\;i\right)$34:    **if** $\varepsilon^{*}>\varepsilon_{i}^{\mathrm{tot}}$ **then**35:        S←S∪{i}36:    **end if**37:
**end for**
38:**return** (fail,S) **if** S≠⌀ **else** (pass,⌀)


## 5. Correctness and Security Analysis

### 5.1. Correctness and Security Statements

**Proposition** **1.** *Completeness and Functional Correctness.*

Under honest execution of Algorithms 1–3, for each query round, the probability that Verify outputs 1 is at least 1−negl(λ), and the accepted record $\left(y_{t},\Delta \varepsilon_{t},\varepsilon_{t},idx_{t},rec_{t}\right)$ is consistent with the public $\left(op_{t},\theta_{t}\right)$ and the mechanism and budget rules given in Section 3.

**Proposition** **2.** *Differential Privacy Guarantee.*

Let view denote the visible outputs and meta-information of a device in a sequence of *T* accepted interactions. Applying accountant Acc to this interaction yields a composition bound (ε,δ). Then for any adjacent input sequences, view satisfies (ε,δ)-DP in the local sense.

**Proposition** **3.** *Unforgeability and k-Use Limitation.*

Under the assumptions of commitment binding, EU-CMA security of digital signatures, VRF pseudorandomness and uniqueness, hash collision resistance, and zero-knowledge proof completeness and soundness, any PPT adversary without knowledge of the device’s internal witness can, with only negligible probability:

(1) Construct a record that is accepted by Verify but inconsistent with the mechanism or budget rules in Section 3;
(2) Cause the number of accepted invocations for a device to exceed its limit $k_{i}$;
(3) Generate a public transcript that passes Algorithm 4 audit but contradicts the online Verify conclusion.

### 5.2. Completeness and Functional Correctness

**Theorem** **1.** *Completeness and Functional Correctness.*

Under honest execution of Algorithms 1–3, for any round *t*, we have

(30) $$Pr\left[\mathrm{Verify}=1\right]\geq 1-negl\left(\lambda \right)$$

and each accepted record satisfies:

Budget update relation:

(31) $$\Delta \varepsilon_{t}=\varepsilon \left(op_{t},\theta_{t}\right),\;\;\varepsilon_{t}=\varepsilon_{t-1}-\Delta \varepsilon_{t}\geq 0$$

The distribution of restricted answer $y_{t}$ is exactly consistent with the local mechanism corresponding to $op_{t}$ (threshold, binning, or prefix mechanism) in Section 3.3. VRF and hash chain relations:

(32) $$\left\{\begin{array}{c} \left(r_{t},\pi_{t}^{\mathrm{vrf}}\right)=VRF.Eval\left(sk_{\mathrm{vrf}},\;\left(i,t\right)\right)\\ idx_{t}=H\left(i\;∥\;t\;∥\;r_{t}\right)\\ rec_{t}=H\left(rec_{t-1}\;∥\;idx_{t}\;∥\;meta_{t}\right) \end{array}\right.$$

**Proof.**  The registration phase (Algorithm 1) primarily establishes the binding relationship between device identity keys $\left(pk_{i},pk_{i}^{vrf}\right)$ and privacy policy parameters $\left(\varepsilon_{tot},k_{i}\right)$. This phase does not involve static commitments to subsequent real-time observation data. □

In the *t*-th restricted query (Algorithm 2), the device independently samples random number $\rho_{t}$ for the current real-time observation value $x_{i,t}$, generating a dynamic commitment:

(33) $$C_{i,t}=\mathrm{Com}\left(x_{i,t};\rho_{t}\right)$$

Based on the computational binding property of the commitment scheme, within polynomial time, an attacker cannot find another distinct input $\left(x^{\prime },\rho^{\prime }\right)$ such that $\mathrm{Com}\left(x^{\prime };\rho^{\prime }\right)=C_{i,t}$ holds. Therefore, the subsequently generated zero-knowledge proof $\pi_{t}$ can strictly constrain that the restricted answer $y_{t}$ is indeed correctly generated from the true value $x_{i,t}$ locked by this commitment $C_{i,t}$ via the specified mechanism.

During the *t*-th query round, Algorithm 2 first calculates the single-round privacy loss and balance according to the rules in Section 3.3:

(34) $$\Delta \varepsilon_{t}=\varepsilon \left(op_{t},\theta_{t}\right),\;\;\varepsilon_{t}=\varepsilon_{t-1}-\Delta \varepsilon_{t}$$

Subsequently, it selects the corresponding local mechanism based on the operation type ${op}_{t}$: when $op_{t}=thresh$, $y_{t}$ is sampled according to the Binary Randomized Response distribution given in Section 3; when ${op}_{t}=bucket$ or prefix, $y_{t}$ is sampled according to the distributions of the *m*-ary Randomized Response mechanism and the Prefix Randomized Response mechanism, respectively. Thus, under honest execution, the distribution of $y_{t}$ is strictly consistent with the corresponding mechanisms in Section 3.

In the same round, Algorithm 2 utilizes the VRF and hash function to compute

(35) $$\left\{\begin{array}{c} \left(r_{t},\pi_{t}^{\mathrm{vrf}}\right)=VRF.Eval\left(sk_{\mathrm{vrf}},\;\left(i,t\right)\right)\\ idx_{t}=H\left(i\;∥\;t\;∥\;r_{t}\right)\\ rec_{t}=H\left(rec_{t-1}\;∥\;idx_{t}\;∥\;meta_{t}\right) \end{array}\right.$$

It then uses these quantities, together with the budget state, operation parameters, and commitment, as inputs and witnesses for the unified relation *R* to construct the ZKP $\pi_{t}$. From the definition of *R* in Section 3.5, under honest execution, we have

(36) $$\left(inp_{t},wit_{t}\right)\in R$$

The ZKP system satisfies completeness over the relation *R*, i.e.,

(37) $$\left(inp_{t},wit_{t}\right)\in R\Rightarrow Pr\left[ZK.Verify\left(PP,R,inp_{t},\pi_{t}\right)=1\right]\geq 1-negl\left(\lambda \right)$$

In Algorithm 3, apart from ZK.Verify, all other checks are direct verifications of the aforementioned budget update equations, VRF, and hash chain equations. Under honest execution, these checks hold true invariably and do not introduce additional rejection events. Consequently, for any round *t*, we have

(38) $$Pr\left[Verify=1\right]\geq 1-negl\left(\lambda \right)$$

Moreover, once Verify=1, the budget update, mechanism distribution, and VRF/hash chain relations are all individually satisfied, thereby guaranteeing functional correctness.

### 5.3. Differential Privacy and Budget Soundness

**Lemma** **1.** *Single-Round Local Differential Privacy.*

Let the operation in round *t* be $\left({op}_{t},\theta_{t}\right)$, and the single-round privacy strength be

(39) $$\Delta \varepsilon_{t}=\varepsilon \left(op_{t},\theta_{t}\right)$$

For any corresponding mechanism *M* (threshold, binning, or prefix) defined in Section 3.3, and for any pair of adjacent inputs $x,x^{\prime }$, and any output *y*, we have

(40) $$\frac{Pr\left[M\left(x;\theta_{t}\right)=y\right]}{Pr\left[M\left(x^{\prime };\theta_{t}\right)=y\right]}\leq e^{\Delta \varepsilon_{t}}$$

**Proof.**  For the binary threshold mechanism, Section 3.3 gives its randomized response parameter as

(41) $$p=\frac{e^{\Delta \varepsilon_{t}}}{e^{\Delta \varepsilon_{t}}+1}$$

□

When the input bit b∈{0,1}:

(42) $$Pr\left[M_{\mathrm{thresh}}\left(b;\theta_{t}\right)=b\right]=p$$

(43) $$Pr\left[M_{\mathrm{thresh}}\left(b;\theta_{t}\right)=1-b\right]=1-p$$

For any output y∈{0,1}, we have

(44) $$\frac{Pr\left[M_{\mathrm{thresh}}\left(0;\theta_{t}\right)=y\right]}{Pr\left[M_{\mathrm{thresh}}\left(1;\theta_{t}\right)=y\right]}\in \left\{\frac{p}{1-p},\frac{1-p}{p}\right\}=\left\{e^{\Delta \varepsilon_{t}},e^{-\Delta \varepsilon_{t}}\right\}\leq e^{\Delta \varepsilon_{t}}$$

Thus, it satisfies $\Delta \varepsilon_{t}$-LDP.

For the binning and prefix mechanisms, Section 3.3 has already established that for any bin index *j*, when the input falls into that bin, the probability of outputting $y_{j}$ is

(45) $$Pr\left[M\left(x;\theta_{t}\right)=y_{j}\right]=\frac{e^{\Delta \varepsilon_{t}}}{e^{\Delta \varepsilon_{t}}+m-1}$$

When the input is not in that bin, the corresponding probability is

(46) $$Pr\left[M\left(x^{\prime };\theta_{t}\right)=y_{j}\right]=\frac{1}{e^{\Delta \varepsilon_{t}}+m-1}$$

where *m* is the number of output values. The ratio between the two is

(47) $$\frac{Pr\left[M\left(x;\theta_{t}\right)=y_{j}\right]}{Pr\left[M\left(x^{\prime };\theta_{t}\right)=y_{j}\right]}=e^{\Delta \varepsilon_{t}}$$

Therefore, it similarly satisfies the Local $\Delta \varepsilon_{t}$-DP condition. Since the above inequality holds uniformly for all three types of mechanisms, the lemma follows.

**Theorem** **2.** *Budget Soundness and Differential Privacy Composition.*

Consider a device involved in *T* rounds of accepted interactions, where the operation in round *t* is $\left({op}_{t},\theta_{t}\right)$, and the single-round loss is

(48) $$\Delta \varepsilon_{t}=\varepsilon \left(op_{t},\theta_{t}\right)$$

Let the output of the differential privacy accountant defined in Equation (17) be

(49) $$\left(\varepsilon_{\mathrm{acc}},\delta_{\mathrm{acc}}\right)\leftarrow Acc\left(\{\left(op_{t},\theta_{t},\Delta \varepsilon_{t}\right){\}}_{t=1}^{T}\right)$$

Then, the overall randomized mapping from the device’s raw observation sequence to the public output sequence satisfies $\left(\varepsilon_{\mathrm{acc}},\delta_{\mathrm{acc}}\right)$-DP in the local sense. Proof. By Lemma 1, for every round *t*, any adjacent inputs $x_{t},x_{t}^{\prime }$, and single-round output $y_{t}$, we have

(50) $$\frac{Pr\left[y_{t}|x_{t}\right]}{Pr\left[y_{t}|x_{t}^{\prime }\right]}\leq e^{\Delta \varepsilon_{t}}$$

Let the entire output sequence be $\left(y_{1},\ldots ,y_{T}\right)$ and the two adjacent input sequences be $\left(x_{1},\ldots ,x_{T}\right)$ and $\left(x_{1}^{\prime },\ldots ,x_{T}^{\prime }\right)$. Given the output sequence, the ratio of the joint probabilities for the entire sequence can be written as the product of the single-round ratios:

(51) $$\frac{Pr\left[\left(y_{1},\ldots ,y_{T}\right)|\left(x_{1},\ldots ,x_{T}\right)\right]}{Pr\left[\left(y_{1},\ldots ,y_{T}\right)|\left(x_{1}^{\prime },\ldots ,x_{T}^{\prime }\right)\right]}=\prod_{t=1}^{T}\frac{Pr\left[y_{t}|x_{t}\right]}{Pr\left[y_{t}|x_{t}^{\prime }\right]}\leq exp\left(\sum_{t=1}^{T}\Delta \varepsilon_{t}\right)$$

The definition of the Accountant Acc in Section 3 ensures that its output $\varepsilon_{\mathrm{acc}}$ covers at least the sum of the aforementioned losses, i.e.,

(52) $$\varepsilon_{\mathrm{acc}}\geq \sum_{t=1}^{T}\Delta \varepsilon_{t}$$

and provides a corresponding upper bound on the failure probability $\delta_{\mathrm{acc}}$, consistent with the (ε,δ) form in the standard Differential Privacy Composition Theorem. Replacing the sum in the exponent of the above equation with $\varepsilon_{\mathrm{acc}}$ yields that the overall mechanism satisfies Local $\left(\varepsilon_{\mathrm{acc}},\delta_{\mathrm{acc}}\right)$-DP for any device.

### 5.4. k-Use Limitation and Audit Replayability

**Lemma** **2.** *Uniqueness of Index and Receipt Chain.*

Assuming the uniqueness of the VRF and the collision resistance of the hash function, for any device identifier *i* and round *t*, once the output $r_{t}$ is obtained via

(53) $$\left(r_{t},\pi_{t}^{\mathrm{vrf}}\right)=VRF.Eval\left(sk_{\mathrm{vrf}},\left(i,t\right)\right)$$

then

(54) $$idx_{t}=H\left(i||t||r_{t}\right)$$

is uniquely determined. Given the previous receipt ${\mathrm{rec}}_{t-1}$ and meta-information ${meta}_{t}$, the recursive definition

(55) $$rec_{t}=H\left(rec_{t-1}||idx_{t}\left|\right|meta_{t}\right)$$

is also uniquely determined. If there exist two different sets of ${idx}_{t}$ or ${rec}_{t}$ for the same (i,t) that still pass verification, one could construct a VRF output reuse or a hash collision, which contradicts the assumption.

**Theorem** **3.** *k-use Soundness and Audit Equivalence.*

Assuming the binding property of commitments, the pseudorandomness and uniqueness of the VRF, the collision resistance of the hash function, and the soundness of the ZKPs, the following hold:

1. If the Online Verification Algorithm (Algorithm 3) returns Verify=1 for all records of a specific device *i*, then that device satisfies the budget update rules and *k*-use limits in all accepted rounds. That is, for every round *t*,
  (56) $$\Delta \varepsilon_{t}=\varepsilon \left(op_{t},\theta_{t}\right),\;\;\varepsilon_{t}=\varepsilon_{t-1}-\Delta \varepsilon_{t}\geq 0$$
  it holds, and the total number of accepted invocations does not exceed the preset upper limit $k_{i}$.
2. For any public transcript *T*, the accept/reject conclusions yielded by the Online Verification Algorithm (Algorithm 3) and the Offline Replay Audit Algorithm (Algorithm 4) for every record in *T* are completely identical.

**Proof.**  First, we prove the first claim. Assume, for the sake of contradiction, that there exists a record that violates the budget update or *k*-use conditions in actual execution but still satisfies Verify=1. Let the public input and real witness corresponding to this record be $\left({inp}_{t},{wit}_{t}\right)$; then, we have

(57) $$\left(inp_{t},wit_{t}\right)\notin R$$

(58) $$ZK.Verify\left(PP,R,\mathrm{in}p_{t},\pi_{t}\right)=1$$

By the knowledge soundness of the ZKP, we can extract another witness ${wit}^{\prime }$ from $\pi_{t}$ such that

(59) $$\left(inp_{t},wit\right)\in R$$

The unified relation *R* explicitly incorporates commitment consistency, budget deduction consistency, and *k*-use conditions. If the actual execution has already violated the budget or invocation limit, then any witness satisfying *R* must inevitably contradict $C_{i,t}=Com\left(x_{i,t};\rho_{t}\right)$ or the equations for ${idx}_{t}$ and ${rec}_{t}$ given in Lemma 2. This implies the construction of an attack that breaks the binding property of the commitment or the security of the VRF/Hash, which contradicts the aforementioned assumptions. Therefore, under these assumptions, the probability of such an event—where a violation occurs yet the verification passes—is at most negl(λ), thereby establishing the soundness of the budget and *k*-use mechanisms. □

Next, we prove the second claim. Given a public transcript *T*, the sequence of checks executed by Algorithms 3 and 4 on each record is completely identical. Both involve verifying the VRF proof and checking:

(60) $$idx_{t}\overset{?}{\;=}H\left(i||t||r_{t}\right)$$

(61) $$rec_{t}\overset{?}{\;=}H(rec_{t-1}||idx_{t}||meta_{t})$$

and reconstructing the budget and *k*-use states according to

(62) $$\Delta \varepsilon_{t}=\varepsilon \left(op_{t},\theta_{t}\right),\;\;\varepsilon_{t}=\varepsilon_{t-1}-\Delta \varepsilon_{t}$$

Finally, both invoke $ZK.Verify(PP,R,{inp}_{t},\pi_{t})$. All the aforementioned judgments rely solely on the public fields in *T* and the states derived from the prefix; they are deterministic computations that do not depend on the device’s internal randomness or private information. Consequently, for the same transcript *T*, the accept/reject conclusions yielded by the two algorithms for each record must be identical.

## 6. Implementation and Experimental Evaluation

### 6.1. Prototype Implementation

Our reference implementation consists of three parts: a device-side library, an edge relay, and a public verifier. The device-side library encapsulates all logic of Algorithms 1 and 2: During registration, it generates device keys and initial receipts; during each query, it generates dynamic commitments for current observations and constructs proofs; during queries, it derives session randomness from a PRF, produces restricted answers via lightweight mechanisms (threshold, binning, or prefix), and packages the joint predicate of mechanism consistency, budget deduction, and k-use into a zero-knowledge proof. Randomization within the library relies only on local PRF and cryptographic-grade pseudorandom sources, without accessing external states. The edge relay is only responsible for forwarding, rate limiting, and buffering; it does not participate in trusted computation nor maintain persistent counters; its message formats in both directions are completely public, facilitating packet capture and replay. The public verifier implements Algorithms 3 and 4 and can replay transcripts offline on any endpoint, reconstruct budget trajectories and receipt chains, and independently render acceptance conclusions.

Cryptographic instantiation follows the principle of choosing the best inside and outside circuits: Commitments use an additively homomorphic scheme for efficient inside circuits; hash functions use constraint-friendly constructions inside circuits and mature collision-resistant algorithms outside circuits; VRF is a standardized implementation for independent deployment and audit reproduction. The proof system only requires completeness, knowledge soundness, and zero-knowledge; the specific backend can be replaced without affecting interfaces and audit processes. All choices are constrained by constant overhead on the device and edge sides, avoiding loops proportional to the size of specific numerical domains.

**Figure 2:**
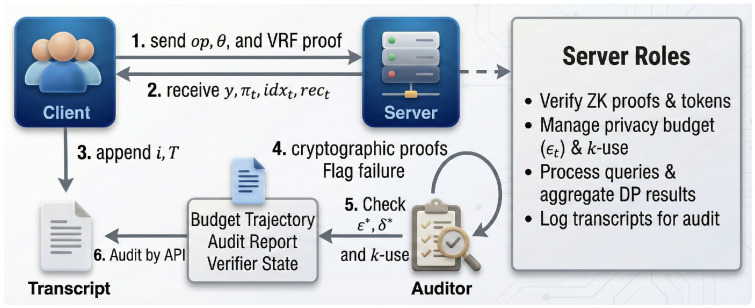
Evidence artifacts from the public transcript: budget trajectory, audit report, and verifier state.

The interaction relationships among system components and the client verification pipeline are shown in Figure 2.

### 6.2. Experimental Environment

Experiments were conducted on a 16-core x86-64 server (3.4 GHz, 64 GB RAM, Ubuntu 22.04, kernel 5.15). The cryptographic layer uses SNARK construction on the BLS12-381 curve; the in-circuit hash uses Poseidon; budget commitment is based on the Pedersen scheme on the Jubjub curve; the session index/randomness source uses ECVRF; and the receipt chain is a hash chain. The system is compiled with Rust 1.75, with OpenSSL 3.x supporting random numbers and hashing; each experiment warms up for 30 s, the sampling window is 3 min, and the results are the median of five independent runs. The cryptographic primitives and components used are detailed in Table 2.

### 6.3. Data and Workload

**Figure 3:**
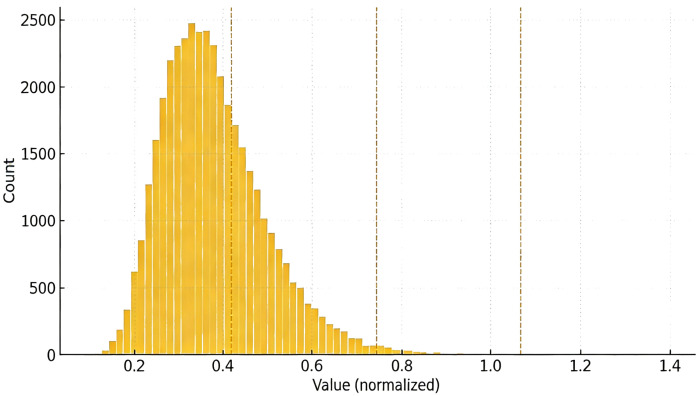
Value distribution with bucket overlays (B = 256 dashed, B = 1024 dotted).

We tested system performance on two de-identified datasets: R-1 with about 1.2 million rows containing non-negative monetary fields and R-2 with about 8.4 million rows containing heavy-tailed usage fields. Additionally, three types of synthetic columns were generated: Gaussian N (0, 1) distribution, log-normal (μ=6,σ=1.2) distribution, and Zipf distribution (range $\left[1,2^{20}\right]$, exponent s=1.1). The value distributions are visualized in Figure 3. Experiments set three operators: threshold, binning, and prefix; number of buckets B∈{256,1024}; prefix length b∈{4,8,12}; and concurrency streams 1, 8, and 16. Each k-use token’s authorization limit is k∈{10,100}. These settings cover typical end-edge scenarios from single stream to high concurrency, as summarized in Table 3.

### 6.4. Evaluation Metrics

System metrics include throughput and p50/p95 end-to-end latency, with separate breakdowns of device-side generation latency and verifier verification latency, allowing proof-related overhead to be separated from network/business overhead. Evidence chain metrics include single receipt size, VRF verification cost, and receipt chain growth rate. Differential privacy accuracy metrics depend on the task: threshold judgment reports ROC and true positive/false positive at target operating points; binning reports macro accuracy and mean absolute bucket error; prefix use case reports prefix matching error rate and re-identification rate under multi-round synthesis. Audit-related metrics report offline replay speed (verification CPU/memory per thousand transcript records) and receipt chain scanning cost under different time window lengths. All charts report the median of five independent runs; error bars are 95% bootstrap confidence intervals (1000 resamples). Latency statistics are sampled after a 30 s warm-up over a 3 min window.

### 6.5. Baselines

To attribute the cost and benefit of evidence generation, evaluation includes three baselines. The first is proof-free local differential privacy, producing only restricted answers without proofs of mechanism consistency and budget deduction, and the verifier does not check receipt chains; this baseline gives the accuracy upper bound and cost lower bound of randomization only. The second is the server-state counting version, where invocation counts are accumulated and rate-limited on a server-side counter without generating receipt chains; it represents a common engineering alternative but cannot be audited offline. The third is differential-privacy-free threshold/binning, only for functional reference, showing the ultimate accuracy without budget constraints. Our method and the three baselines are aligned on the same task, same parameters, and same network conditions, reporting differences in throughput, latency, evidence size, and verification cost, allowing readers to directly see the marginal cost from claim to evidence. A summary comparison of these methods is provided in Table 4.

### 6.6. Experimental Procedure

Each experiment fixes a task, an output domain, and a privacy/invocation budget configuration. The system first completes registration, generating commitments and registering public metadata, and then enters multiple invocation rounds, each producing a restricted answer and proof, delivered via the edge relay to the verifier. The verifier performs online acceptance, recording whether it passed and various latencies; after the experiment, an offline audit is run immediately, replaying Algorithm 3 based on the transcript, reconstructing budget trajectories, and checking whether balances are consistent with online records. Privacy parameters are scanned over a preset grid, covering low, medium, and high ε configurations; bin categories from small to medium range; and prefix lengths from very short to medium. Invocation limit *k* takes several typical values and combines with the privacy budget to form a dual-gate strategy. To avoid cache and warm-up effects affecting results, all curves are sampled in stable phases.

### 6.7. Results Interpretation

Under end/edge constraints, proof generation and verification overhead are constant-level, mainly determined by the fixed gate count of the chosen proof system, with no linear relationship to input domain size. The p95 end-to-end latency is less sensitive to ε; the impact of bin category count and prefix length mainly comes from the fixed overhead of encoding and proof circuits. Compared with the proof-free local differential privacy baseline, our method shows a stable but predictable constant-factor increase in throughput and latency; compared with the server-state counting baseline, our method has similar online throughput, but offline audit accessibility is significantly stronger, and cross-node replay does not require access to server historical states. Evidence size grows linearly with invocation count, but the slope is dominated by receipt chain digest length and does not expand with data domain size; single-core verification speed on the audit side is approximately linear with workload scale, decoupled from the number of edges and devices, making large-scale offline audits computationally and storage feasible. As shown in Table 5, replay audit throughput reaches about 23 k–25 k QPS. This high performance, about 15 times the single verification speed, is directly attributed to the batch verification strategy.

**Figure 4:**
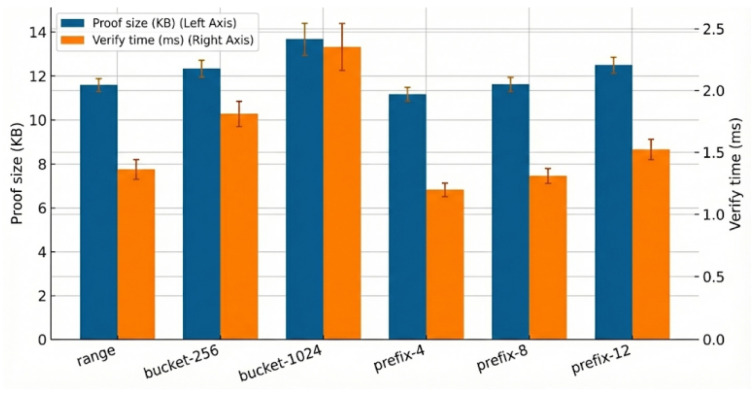
Microbenchmark: proof size and verify time (95% CI).

To more intuitively observe the performance overhead of different operators in a single operation, we measured their proof sizes and verification times, as shown in Figure 4.

Results show that the prefix series has significantly lower overhead on the verification side than the bucket series, while bucket-1024 has the highest constant term in proof size. Overall, the cost fluctuations of each operator remain at the millisecond level and mainly vary with circuit structure, unaffected by specific data scale.

**Energy Consumption and Feasibility Analysis**

**Figure 5:**
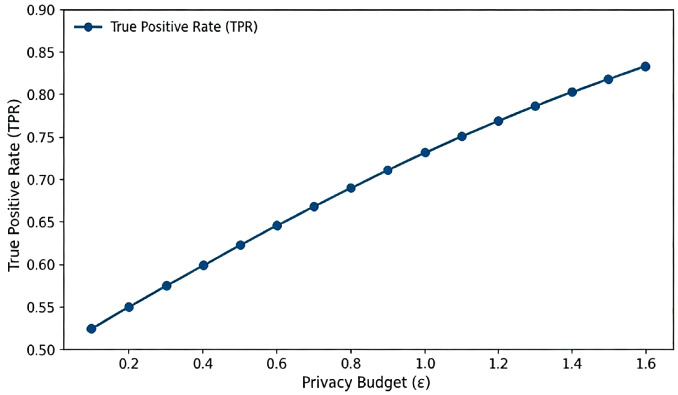
Impact of privacy budget ε on utility and performance.

Given the sensitivity of IoT devices to battery life, we further evaluated the energy consumption overhead of the scheme based on measured latency data and a standard hardware power model. According to our microbenchmark tests (as shown in Figure 5), the proof generation latency for a single restricted query remains stable at around 10.8 ms. Referring to energy consumption benchmark studies for similar embedded platforms (ESP32-WROOM-32, main frequency 240 MHz) [30], at a working voltage of 3.3 V and average working current of 160 mA, the estimated energy consumption for this level of computational task is approximately 5.7 mJ.

As a comparison, existing IoT communication energy consumption research [31] indicates that the energy consumption for establishing a connection via Wi-Fi or NB-IoT and sending a single standard MTU data packet is typically between 100 mJ and 300 mJ and is significantly affected by signal quality. This shows that the computational energy consumption introduced by this scheme is only 2% to 5% of the single wireless transmission energy consumption. Therefore, the “Computation-for-Communication” strategy adopted in this scheme—i.e., compressing high-dimensional raw data into constant-size zero-knowledge proofs and short receipts through local computation—has significant advantages in reducing radio active time, demonstrating its engineering feasibility in battery-powered constrained environments.

**Figure 6:**
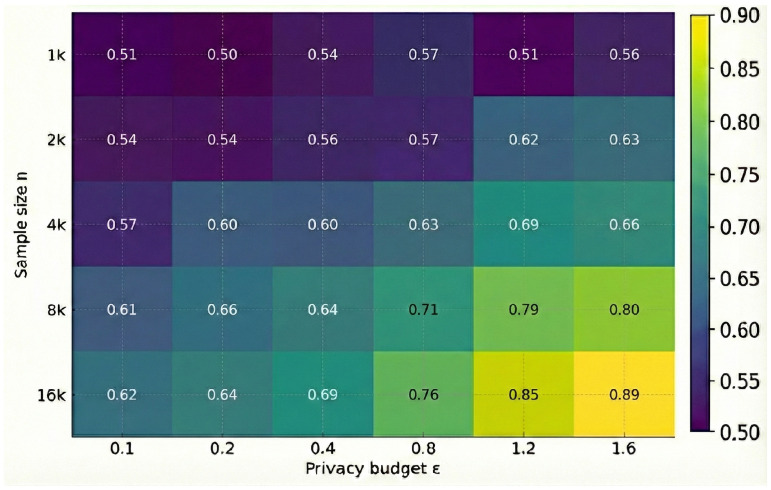
Threshold task utility: TPR vs. (ε,n).

In terms of accuracy, threshold tasks maintain stable ROC shapes with adequate separation between true and false positives, as shown in Figure 6. Binning tasks shows controllable mean absolute bucket errors within medium category ranges, while prefix lengths should be restricted to mitigate potential linkage risks with binning. Experiments on collusion and splicing attacks demonstrate that re-identification rates are effectively constrained by the dual-gate mechanism; queries are rejected once the cumulative leakage reaches the audit bound, consistent with the theoretical analysis in Section 5.

To provide quantitative guidance for parameter configuration in actual deployment, we further evaluated the sensitivity of privacy budget ε in the range [0.1, 1.6] under threshold judgment tasks (as shown in Figure 5). Experiments found that proof generation latency is significantly insensitive to changes in privacy budget, remaining constant across the evaluated range. Theoretically, this is because adjusting ε only changes the public input values of the circuit, without altering the number of gate constraints or the topology of the circuit, so the device-side FFT and MSM computational load remains constant. In contrast, the true positive rate of data shows the expected sigmoid growth pattern: In the strong privacy region ε<0.4, TPR is close to random guessing, while in the ε∈[0.8,1.2] range, TPR rapidly climbs to the highly usable range of 0.70–0.77.

**Figure 7:**
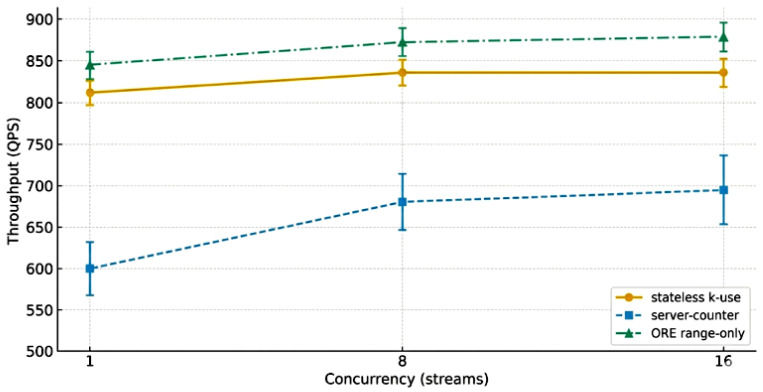
Throughput across concurrencies (95%CI).

In end-to-end scenarios, we further compared the throughput performance of different operation modes under different concurrency scales, as shown in Figure 7 and Table 6.

**Figure 8:**
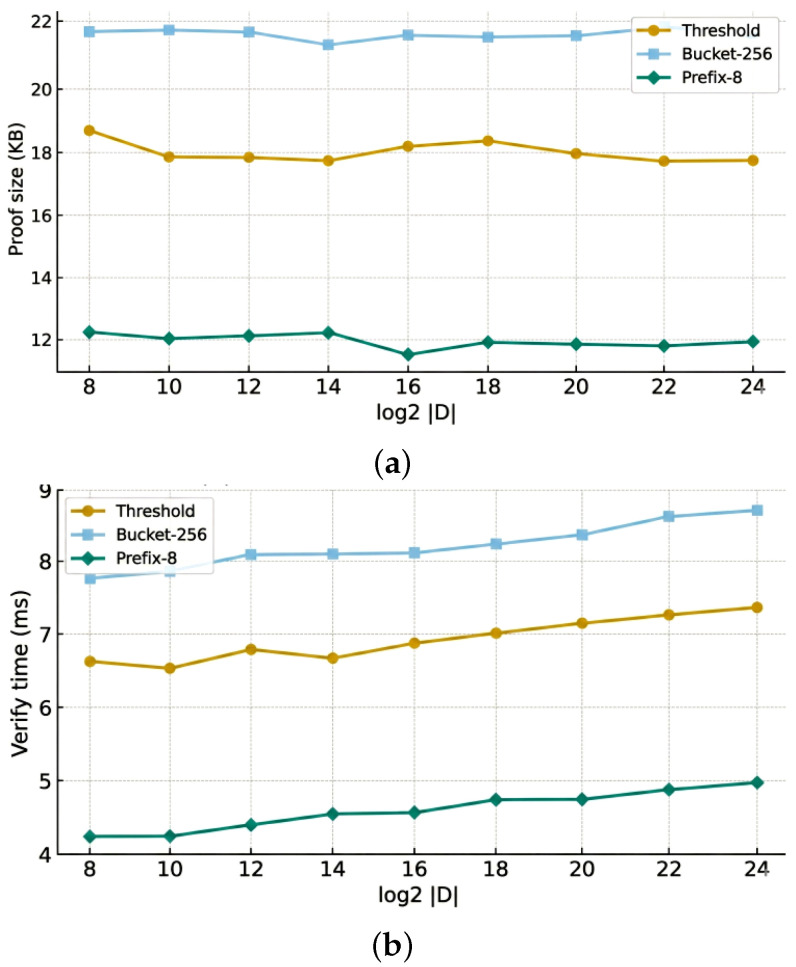
Analysis of proof size and verification time versus domain size: (**a**) proof size; (**b**) verify time.

It can be seen that as concurrency increases from 1 to 8 and 16, the throughput improvement of server-counter mode is more significant, while stateless k-use and ORE range-only mainly maintain a more stable high plateau range. Overall, each mode maintains expected scalability as concurrency grows, with no obvious bottlenecks. Additionally, a detailed analysis of proof size and verification time versus domain size is provided in Figure 8.

### 6.8. Performance-Utility Trade-Off Analysis and Hybrid Mechanism Exploration

To further validate the rationale for selecting the Randomized Response (RR) mechanism on resource-constrained IoT devices and explore optimization paths for high-dimensional query scenarios, this section conducts a quantitative trade-off analysis between the RR and Optimized Local Hashing (OLH) mechanisms. The experiment sets a privacy budget of ε=1.0, a sample size of n=10,000, and selects four typical bucket sizes B∈{16,64,256,1024}, focusing on two core metrics: The Mean Squared Error (MSE) reflecting data utility and the zero-knowledge proof generation time (Proving Time) reflecting computational overhead.

**Figure 9:**
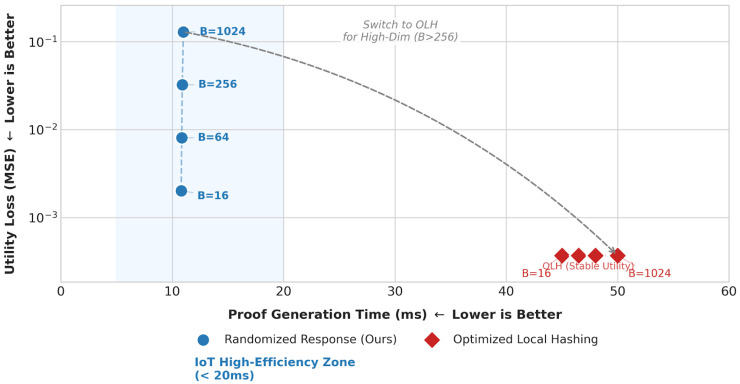
Performance-utility trade-off analysis.

As shown in Figure 9, the experimental results exhibit significant Pareto trade-off characteristics:

**Performance Dimension:** The RR mechanism (blue dots) adopted in this paper demonstrates extremely high computational efficiency, with proof generation time stabilizing around 11 ms, showing almost no fluctuation as the bucket count *B* increases. In contrast, the OLH mechanism (red dots), due to its reliance on cryptographic hash functions such as Poseidon, incurs a significantly higher number of constraints in arithmetic circuits compared with the simple Boolean operations of RR, leading to proof generation times as high as 45–50 ms, approximately 4–5 times that of RR.

**Utility Dimension:** As *B* increases from 16 to 1024, the MSE of RR shows a linear growth trend, indicating that its utility is indeed limited when publishing high-dimensional data; whereas the MSE of OLH remains at a low and stable level, unaffected by dimensionality.

The experimental data clearly delineate an “IoT High-Efficiency Zone”. In the most common low-dimensional, high-frequency query scenarios in IoT (such as threshold judgments with B<64 or coarse-grained state classification), the RR mechanism achieves a several-fold performance improvement at the cost of minimal utility loss, making it the optimal solution under current engineering conditions.

Based on this analysis, we propose a future evolution direction of a “Hybrid Mechanism”: the system can dynamically switch mechanisms based on the dimensionality of the query request. When B≤256, RR is prioritized to ensure real-time performance and low energy consumption; when B>256, the system automatically switches to the OLH mechanism to maintain the statistical utility of high-dimensional data. Thanks to the unified verification relation R proposed in this paper, this switching can be seamlessly implemented without altering the underlying verification architecture.

### 6.9. Related Work Comparative Analysis

To clarify the position of our scheme in existing verifiable differential privacy research, we select representative recent works for quantitative comparison from two dimensions: verification time and proof system scale. The comparison results are summarized in Table 7 and Table 8. Our work builds upon foundational zero-knowledge proof systems [32,33] and range proof techniques [34], extending them to support verifiable differential privacy operations in IoT settings.

### 6.10. Threats and Limitations

Experiments do not cover device source value forgery and physical side-channel leakage; these risks require other layers of measures. Network simulation can only approximate congestion and packet loss distributions in real operational environments, possibly underestimating tail latency in extreme cases. The proof backend and primitive implementation can be replaced; different choices change constant factors and engineering performance but do not alter verification semantics and audit processes.

## 7. Conclusions and Future Work

Starting from the engineering reality of IoT end-edge-cloud collaboration, this paper proposes a unified framework of “restricted answers + verifiable evidence”. On the end/edge side, we use lightweight randomization mechanisms to generate restricted answers for operators such as threshold judgment, binning statistics, and prefix disclosure. At the policy level, we introduce auditable privacy budgets and k-use upper limits, forming dual gates: constraining invocation counts and cumulative leakage. At the evidence level, we combine commitments, hashes, and VRF-derived indexes to construct chained receipts, compressing each accepted invocation into a publicly replayable evidence entry. At the verification level, a unified verification relation links mechanism consistency, budget deduction consistency, and invocation limit consistency, enabling any party to independently check compliance without accessing raw data and supporting transcript-based offline audits.

Based on these results, three conclusions can be drawn: First, unifying externally visible behaviors into a single verification relation can transform “whether privacy/invocation policies are followed” into a machine-auditable factual judgment, thereby weakening dependence on server persistent states and trusted hardware. Second, under practical privacy budgets and medium output domains, the constant overhead of proofs and receipt chains is mainly controlled by the proof system gate count and hash digest length, approximately decoupled from specific data domain scale, making large-scale offline audits feasible in both computation and storage. Third, by combining deductible privacy budgets and k-use limits into dual gates, the feasible window for cross-subject, cross-round splicing attacks can be explicitly defined, providing regulators and data providers with a clear “splicing upper bound.”

Looking ahead, there are three directions worth further exploration: First, introducing adaptive accounting and policy tuning during operation so that the budget cost per invocation and the precision required by business can be automatically calibrated. Second, exploring more proof backends and hash/VRF combinations for different hardware and deployment environments, reducing end/edge latency and resource consumption while maintaining semantic equivalence. Third, extending to multi-attribute and temporal features while keeping the unified verification relation semantics unchanged, supporting more complex composite threshold, binning, and prefix operators, and overlaying with centralized statistical aggregation mechanisms, providing infrastructure for cross-organization data collaboration that meets compliance requirements and possesses verifiable evidence chains.

Overall, this paper transforms platform commitments into evidence obligations and privacy boundaries into machine-auditable composition upper bounds, providing a robust engineering path for cross-organization data collaboration in IoT. We hope this method can form a positive cycle with industry compliance requirements, standardization efforts, and open-source ecosystems, promoting the practice of minimal usable information + verifiable evidence to become the new norm for IoT data sharing.

## Figures and Tables

**Table 1:** Notation and public parameters.

Parameter	Description
λ	Security parameter for cryptographic primitives
*i*	IoT device identifier
t∈N	Invocation round index for the device
X⊂R	Domain of a single observation
$x_{i,t}\in X$	Scalar observation of device *i* at round *t*
PP	Public parameters
$o_{t}$	Operation type at round *t*
$\theta_{t}$	Parameters depending on $o_{t}$
$y_{t}$	Output after local differential privacy perturbation
$\varepsilon (o_{t},\theta_{t})$	Single-round privacy strength, also denoted $\Delta \varepsilon_{t}$
$\varepsilon_{\mathrm{tot}}$	System-preset total privacy budget for the device
$\varepsilon_{t}$	Remaining privacy budget balance (initial $\varepsilon_{0}=\varepsilon_{\mathrm{tot}}$,
	decreasing with invocations)
Acc	Accounting function, outputs composition bound (ε,δ) given history
(ε,δ)	Record satisfies (ε,δ)-DP under all accepted invocations
*k*	Maximum number of successful invocations per device
H(·)	Hash function for generating indexes and receipt chains
$r_{t},\pi_{t}^{\mathrm{vrf}}$	Computed on input (i,t) via VRF
$idx_{t}$	Session index for the *t*-th invocation
$meta_{t}$	Metadata uniquely identifying the *t*-th invocation
$rec_{t}$	Receipt chain
*T*	Public transcript
ZK.Prove	Proof algorithm of the zero-knowledge proof system
ZK.Verify	Verification algorithm of the zero-knowledge proof system
$\pi_{t}$	Zero-knowledge proof for the *t*-th invocation
$\eta_{t}$	Noise source for generating $y_{t}$
$wit_{t}$	Witness
$inp_{t}$	Public input
$R_{\mathrm{mech}}$	Mechanism consistency
$R_{\mathrm{budget}}$	Budget deduction consistency
$R_{\mathrm{kuse}}$	Invocation limit and receipt consistency
*R*	Unified verification relation
$sk_{\mathrm{vrf}}$	Secret key for VRF evaluation (kept private by device)
$pk_{\mathrm{vrf}}$	Public key for VRF verification (included in PP or registered)
$\left(pk_{i},sk_{i}\right)$	Digital signature key pair of device *i*
$\left(pk_{i}^{vrf},sk_{i}^{vrf}\right)$	Verifiable Random Function (VRF) key pair of device *i*
$C_{i,t}$	Dynamic commitment for round *t*, defined as $\mathrm{Com}(x_{i,t};\rho_{t})$
$\sigma_{t}$	Digital signature for source authentication in round *t*
$\rho_{t}$	Randomness used for the dynamic commitment in round *t*
$Trans_{t}$	Complete transmission package sent to the verifier, containing
	$\left(\sigma_{t},\pi_{t}^{vrf},r_{t},\pi_{t},inp_{t}\right)$

**Table 2:** Cryptographic primitives and components.

Primitive/Component	Instance	Version
Curve	BLS12-381	v1.0
Hash	Poseidon2-128 [24]	v1.1
Commitment	Pedersen (Jubjub [25]) [26]	v1.0
VRF	ECVRF (RFC9381 [27])	v1.0
ZK backend	PLONK-ish w/lookup [28]	v0.9
Signature	Ed25519 [29]	v1.0

**Table 3:** Datasets and task configurations.

Dataset	Size	Distribution	Task	Parameters
R-1	1.2 M	Right-skew monetary	Threshold	ε∈[0.1,1.6],n∈[1k,16k]
R-2	8.4 M	Heavy-tailed usage	Bucket	B∈{256,1024}
Syn-Gauss	1.0 M	*N* (0, 1), rounded	Prefix	b∈{4,8,12}
Syn-LogN	1.0 M	log *N* (*μ* = 6, *σ* = 1.2)	Bucket	B∈{256,1024}
Syn-Zipf	1.0 M	Zipf (*s* = 1.1)	Threshold	ε∈[0.1,1.6],n∈[1k,16k]

**Table 4:** Comparison of methods.

Method	Stateless Verifier	Auditable Replay	Throughput	Privacy/Functional Notes
Proposed	Yes	Yes	High	Upper-bounded $\left(\varepsilon^{*},\delta^{*}\right)$, k-enforced
LDP-only	Yes	No	High	ε tracked locally; no cross-party audit
Server-counter	No	Partial	Medium	Depends on server trust; k enforced centrally
No-DP range-only	Yes	Yes	Highest	Utility high but privacy absent

**Table 5:** Audit replay performance.

Window (Records)	Replay QPS	CPU (Cores)	Memory (MiB)	Fail: Chain Break	Fail: Budget Overflow	Fail: k Overuse
1000	25,002	3.9	124	0.16%	0.09%	0.06%
10,000	24,906	4.7	139	0.18%	0.06%	0.04%
100,000	23,271	5.5	156	0.18%	0.08%	0.07%
1,000,000	22,817	6.3	168	0.22%	0.08%	0.06%

**Table 6:** Performance comparison under different concurrency levels.

Conc.	Stateless k-Use QPS (±CI)	p95 Latency (ms, ±CI)	Server-Counter QPS (±CI)	p95 Latency (ms, ±CI)
1	820 ± 15	78 ± 3	600 ± 20	85 ± 4
8	860 ± 18	112 ± 5	680 ± 22	136 ± 7
16	860 ± 19	122 ± 5	695 ± 24	154 ± 8

**Table 7:** Verification time comparison.

Method	Configuration	Proof Verification Time (ms)
Ours	Threshold	1.41
	Bucket	1.73
	Prefix	1.35
Paper 1 [35]	Single RR	2.26
Paper 2 [36]	Geolife Base	3.454
	Geolife Expand	4.425
	Geolife Shuffle	2.680
	Smart-meter Base	3.484
	Smart-meter Expand	3.540
	Smart-meter Shuffle	2.659
Paper 3 [37]	$\pi_{\xi }$	1.04
	$\pi_{\delta }$	0.961
Paper 4 [38]	Groth16 (JS)	800
	Groth16 (Rapidsnark)	800

**Table 8:** Constraint scale and key material comparison.

Method	Configuration	Constraints	Proving Key Size	Verification Key Size
Ours	Range	64,062	25.4 MB	726 B
	Bucket-256	76,219	29.8 MB	914 B
	Bucket-1024	98,831	41.7 MB	1268 B
	Prefix-4	61,922	24.3 MB	756 B
	Prefix-8	73,454	28.6 MB	898 B
	Prefix-12	88,837	37.6 MB	1004 B
Paper 2 [36]	Geolife Base	55,884	16.1 MB	776 B
	Geolife Expand	74,322	23.4 MB	824 B
	Geolife Shuffle	173,460	53.2 MB	728 B
	Smartmeter Base	56,903	16.3 MB	776 B
	Smartmeter Expand	75,341	23.7 MB	824 B
	Smartmeter Shuffle	174,095	53.3 MB	728 B
Paper 3 [37]	$\pi_{\xi }$	53,404	—	—
	$\pi_{\delta }$	16,977	—	—
Paper 4 [38]	Groth16	5997	3.4 MB	3.5 KB

## Data Availability

Data will be made available on request.

## References

[1] Xiao Y., Jia Y., Liu C., Cheng X., Yu J., Lv W. (2019). Edge computing security: State of the art and challenges. Proc. IEEE.

[2] Al-Garadi M.A., Mohamed A., Al-Ali A., Du X., Ali I., Guizani M. (2020). A survey of machine and deep learning methods for internet of things (IoT) security. IEEE Commun. Surv. Tutor..

[3] Abadi M., Chu A., Goodfellow I., McMahan H.B., Mironov I., Talwar K., Zhang L. (2016). Deep learning with differential privacy. 2016 ACM SIGSAC Conference on Computer and Communications Security (CCS).

[4] Dwork C., McSherry F., Nissim K., Smith A. (2006). Calibrating noise to sensitivity in private data analysis. Theory of Cryptography Conference (TCC).

[5] Dwork C., Roth A. (2014). The algorithmic foundations of differential privacy. Found. Trends Theor. Comput. Sci..

[6] Erlingsson Ú., Pihur V., Korolova A. (2014). RAPPOR: Randomized aggregatable privacy-preserving ordinal response. 21st ACM Conference on Computer and Communications Security (CCS).

[7] Boldyreva A., Chenette N., Lee Y., O’Neill A. (2009). Order-preserving symmetric encryption. Advances in Cryptology–EUROCRYPT 2009.

[8] Chenette N., Lewi K., Weis S.A., Wu D.J. (2016). Practical order-revealing encryption with limited leakage. 23rd Annual Network and Distributed System Security Symposium (NDSS).

[9] Krawczyk H., Rabin M., Mironov Y. (2016). On the security of ORE: Attacks and improvements. Advances in Cryptology–CRYPTO 2016.

[10] Fleischhacker N., Simkin M. (2021). Robust property-preserving hash functions for Hamming distance and more. Annual International Conference on the Theory and Applications of Cryptographic Techniques (EUROCRYPT 2021).

[11] Curtmola R., Garay J., Kamara S., Ostrovsky R. (2006). Searchable symmetric encryption: Improved definitions and efficient constructions. 13th ACM Conference on Computer and Communications Security (CCS).

[12] Naveed M. (2015). The fallacy of composition of OPE and leakage attacks. 24th USENIX Security Symposium.

[13] Cash D., Grubbs P., Perry J., Ristenpart T. (2017). Leakage-abuse attacks against searchable encryption. 2017 IEEE Symposium on Security and Privacy (S&P).

[14] Yang M., Lyu L., Zhao J., Zhu T., Lam K.-Y. (2020). Local differential privacy and its applications: A comprehensive survey. Comput. Netw..

[15] Chaum D., van Heyst E. (1991). Group signatures with controllable linkability. Advances in Cryptology–EUROCRYPT 1991.

[16] Li J., Li N. (2020). Mitigating unauthorized accesses in IoT via rate-limiting credentials. IEEE Internet Things J..

[17] Walfish M., Blumberg A.J. (2015). Verifying computations without reexecuting them. Commun. ACM.

[18] Parno B., Howell J., Gentry C., Raykova M. (2013). Pinocchio: Nearly practical verifiable computation. 2013 IEEE Symposium on Security and Privacy (S&P).

[19] Gabizon A., Williamson Z.J., Ciobotaru O. (2019). PLONK: Permutations over Lagrange-Bases for Oecumenical Noninteractive Arguments of Knowledge.

[20] Micali S. (1999). Verifiable random functions. 40th Annual Symposium on Foundations of Computer Science (FOCS).

[21] Shi R., Wei L., Zhang L. (2024). More Efficient and Verifiable Privacy-Preserving Aggregation Scheme for Internet of Things-Based Federated Learning. Appl. Sci..

[22] Kairouz P., Oh S., Viswanath P. The composition theorem for differential privacy. Proceedings of the International Conference on Machine Learning (ICML).

[23] Mironov I. (2017). Rényi differential privacy. 2017 IEEE 30th Computer Security Foundations Symposium (CSF).

[24] Grassi L., Khovratovich D., Rechberger C., Roy A., Schofnegger M. (2021). Poseidon: A new hash function for zero-knowledge proof systems. 30th USENIX Security Symposium.

[25] Bellés-Muñoz M., Whitehat B., Baylina J., Daza V., Muñoz-Tapia J.L. (2021). Twisted Edwards elliptic curves for zero-knowledge circuits. Mathematics.

[26] Pedersen T.P. (1991). Non-interactive and information-theoretic secure verifiable secret sharing. Advances in Cryptology—CRYPTO 1991.

[27] Goldberg S., Reyzin L., Papadopoulos D., Včelák J. (2023). Verifiable Random Functions (VRFs), RFC 9381. https://datatracker.ietf.org/doc/rfc9381/.

[28] Gabizon A., Williamson Z.J. (2020). Plookup: A Simplified Polynomial Protocol for Lookup Tables. https://eprint.iacr.org/2020/315.pdf.

[29] Bernstein D.J., Duif N., Lange T., Schwabe P., Yang B.-Y. (2012). High-speed high-security signatures. J. Cryptogr. Eng..

[30] Sanchez-Gomez J.A., Gallego-Garcia D., Sanchez-Iborra R., Ruiz-Mas J., Skarmeta A.F. (2018). A Practical Evaluation on RSA and ECC-Based Cipher Suites for IoT High-Security Energy-Efficient Fog and Mist Computing Devices. Sensors.

[31] Andres-Maldonado A., Ameigeiras P., Prados-Garzon J., Ramos-Munoz J.J., Lopez-Soler J.M. (2020). Dissecting Energy Consumption of NB-IoT Devices Empirically. IEEE Sensors J..

[32] Groth J. (2016). On the size of pairing-based non-interactive arguments. Annual International Conference on the Theory and Applications of Cryptographic Techniques (EUROCRYPT).

[33] Gennaro R., Gentry C., Parno B., Raykova M. (2013). Quadratic span programs and succinct NIZKs without PCPs. Annual International Conference on the Theory and Applications of Cryptographic Techniques (EUROCRYPT).

[34] Bünz B., Bootle J., Boneh D., Poelstra A., Wuille P., Maxwell G. (2018). Bulletproofs: Short proofs for confidential transactions and more. 2018 IEEE Symposium on Security and Privacy (S&P).

[35] Bell-Clark J., Gascón A., Li B., Raykova M., Chowdhury A.R. (2025). Verity: Verifiable Local Differential Privacy. https://eprint.iacr.org/2025/851.pdf.

[36] Bontekoe T., Asghar H.J., Turkmen F. (2024). Efficient Verifiable Differential Privacy with Input Authenticity in the Local and Shuffle Model. https://crysp.petsymposium.org/popets/2025/popets-2025-0076.pdf.

[37] Davidow D.M., Manevich Y., Toch E. (2023). Privacy-Preserving Transactions with Verifiable Local Differential Privacy. https://eprint.iacr.org/2023/126.pdf.

[38] Garrido G.M., Sedlmeir J., Babel M. (2022). Towards Verifiable Differentially-Private Polling.

